# Effect of salt stress on the activity of bromelain in pineapple plants grown *In vitro*

**DOI:** 10.1186/1753-6561-8-S4-P199

**Published:** 2014-10-01

**Authors:** Jaci Vilanova-Neta, Diego Menezes, Márcio Barreto, Jaqueline Souza, Antônio Carvalho-Neto, Ana Lédo, Denise Ruzene, Daniel Silva

**Affiliations:** 1 Universidade Tiradentes, Instituto de Tecnologia e Pesquisa, Aracaju, SE, Brasil; 2 Embrapa Tabuleiros Costeiros, Aracaju, SE, Brasil; 3 Universidade Federal de Sergipe, Núcleo de Engenharia de Produção, São Cristovão, SE, Brasil

## Background

Bromelain is a collective name for proteolytic enzymes or proteases (natural proteolytic enzymatic complex) found in tissues including stems, fruit and leaves of the pineapple (*Ananas comosus var. comosus*) and of other plant species of the family Bromeliaceae [1]. Is a bioactive agent possessing remarkable therapeutic properties such as reversible inhibition of platelet aggregation, relief from bronchitis, sinusitis and enhanced absorption of drugs, particularly of antibiotics [2]. Although Brazil is a major producer of pineapple, occupying the first position in 2010 worldwide with a production of 1.5 million tons of fruit, the salt stress, deleterious alterations observed in plants grown in saline conditions, which occur due to intoxication by ions and decline of supply of water and nutrients to the plant [3] is main factor limiting growth and productivity of plants since it causes metabolic responses in plants, affecting and compromising all important processes such as photosynthesis, changes in levels and protein synthesis and activity enzymes, as well as in the synthesis of lipid metabolism and energy [4]. Thus, the objective of this research was to evaluate the influence of salt stress on the activity of bromelain in pineapple plants (*Ananas comosus L. Merril*) cv. Pérola cultured *in vitro*.

## Methods

The bromelain activity was avaliable from crude extracts of leaves and stems from plants of pineapple, *Ananas comosus var. comosus*, cv. Pérola, cultivated *in vitro *in culture medium MS [5] with growth regulator, BAP (6-benzilaminopurina) and ANA (ácido naftalenoacético) in fixed concentrations and sodium chloride at different concentrations, constituting thus, different tretaments. The enzyme was obtained from crude extracts of leaves and stems from plants of pineapple *in vitro*, after mixture in 2.5 ml of 0.2 M phosphate buffer at pH 5.7 and filtering twice through cheesecloth. Total protein concentration was determined by the Bradford method using Coomassie Blue G250 and the proteolytic activity of the bromelain enzyme was determined based on the method described earlier [6,7]. A Bovine Serum Albumin was used as a substrate to be hydrolysed by the enzyme bromelain.

## Results and conclusion

According to the results, the bromelain activity, total protein concentration and specific activity obtained in pineapple plants cv. Pérola showed variation in relation to salinity levels and plant tissue analysis (stem or leaf). In quantitative terms, the more significant levels of proteoltic activity of bromelain were obtained in tissues from pineapple stems of the treatment corresponding to a concentration of 100 mM NaCl. Our findings corroborate results obtained in other vegetables and can be used in optimizing crop when in coastal regions or in saline soils.
